# Chylothorax and constrictive pericarditis in a woman due to generalized lymphatic anomaly: a case report

**DOI:** 10.1186/s13019-018-0752-3

**Published:** 2018-06-05

**Authors:** Hongchun Du, Mai Xiong, Huai Liao, Yifeng Luo, Huijuan Shi, Canmao Xie

**Affiliations:** 1 Department of Respiratory Medicine, The First Affiliated Hospital, Sun Yat-sen University, No 58, Zhongshan 2nd Road, Guangzhou, 510080 China; 2 Department of Cardiac Surgery, The First Affiliated Hospital, Sun Yat-sen University, Guangzhou, China; 3 Department of Pathology, The First Affiliated Hospital, Sun Yat-sen University, Guangzhou, China

**Keywords:** Chylothorax, Constrictive pericarditis, Generalized lymphatic anomaly

## Abstract

**Background:**

Generalized lymphatic anomaly (GLA) is characterized by diffuse or multicentric proliferation of dilated lymphatic vessels resembling common lymphatic malformations. Compared with soft tissue or bone involvement, thoracic involvement may be associated with a worse prognosis.

**Case presentation:**

We reported a case of GLA with chylothorax and constrictive pericarditis in a 29-year-old woman. This patient exhibited remarkable features, including a continuously hemorrhagic chylothorax, constrictive pericarditis, and involvement of bone and neck lymph nodes. After attempting to manage her condition with conservative treatment, the patient underwent pericardial stripping surgery. Exploration revealed abundant hyperplasia of tubular tissue in the aortopulmonary window in both pleural cavities.

**Conclusions:**

This case highlights the importance of maintaining the clinical suspicion of GLA during the follow-up of chylothorax patients. Aggressive pericardial surgery, which is important for both diagnosis and treatment, should be performed in patients with GLA with constrictive pericarditis.

## Background

Chylothorax, the accumulation of chyle in the pleural space, can result in significant respiratory morbidity and immunodeficiency. There are several causes of chylothorax, including trauma, tumors,congenital diseases and granulomatous infections,among others. GLA is a rare disease characterized by diffuse or multicentric proliferation of dilated lymphatic vessels resembling common lymphatic malformation [1]. It is also typically responsible for chylothorax. Although GLA is different from Gorham–Stout disease, kaposiform lymphangiomatosis and lymphangiectasis, proper diagnosis is still difficult. Here, we present a case of GLA with chylothorax and constrictive pericarditis in a 29-year-old woman. The purpose of this paper is to describe a patient with rapidly progressing GLA. Clinicians should exclude lymphatic anomalies whenever nonmalignant thoracic disorders are found. Aggressive pericardial surgery should be performed in GLA cases with constrictive pericarditis (CP),and this procedure is important for both diagnosis and treatment.

## Case presentation

**Fig. 1:**
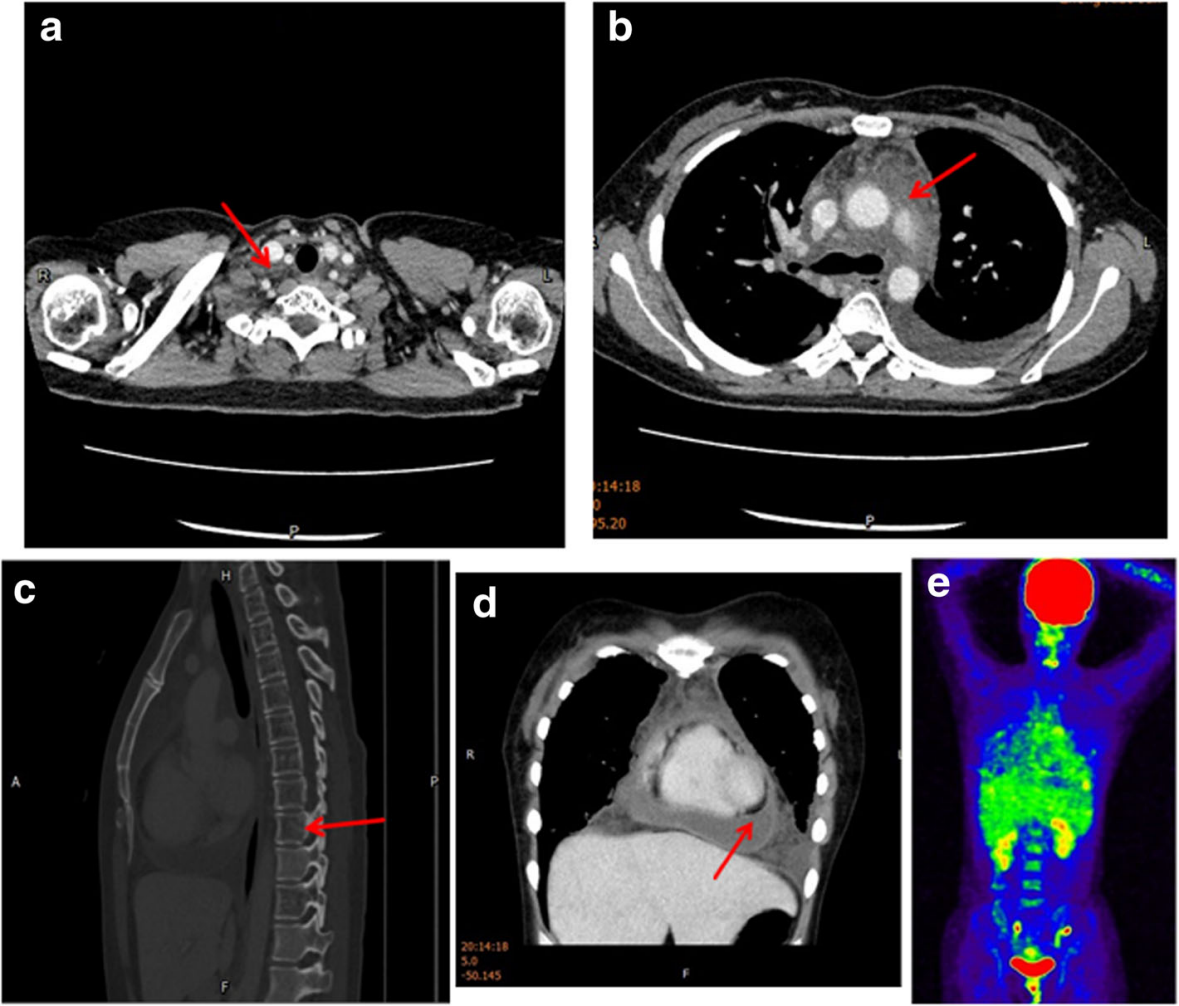
Preoperative chest images (**a**) Cross –sectional CT image shows enlargement of lymph nodes (arrow) on the right carotid artery. **b** CT image reveals soft tissue density shadow in the mediastinum (arrow) in place of adipose tissue, with mild reinforcement (CT value = 32). **c** Thoracic sagittal section CT image shows diffuse reduction of the intensity in C7, T1-T8 (the arrow points to T8), T10 bodies, and bone absorption is indicated. **d** Thoracic coronal sectionCT image shows pericardial effusion and pleural effusion. **e** PET-CT image shows slightly increased FDG uptake in the region of neck and mediastinum

Written informed consent for publication of this case report, accompanying images, and any additional related information was obtained from the patient. A 29-year-old woman from a coastal city in South China was admitted to our hospital on October 9th, 2016. The patient had been complaining of polypnea, cough and chest tightness for approximately 20 days. Her social, occupational and family histories were unremarkable. The patient had no medical and surgical histories. On physical examination, she showed sinus tachycardia and generalized cardiac enlargement determined by percussion. All other signs were negative. Chest CT showed pericardial effusion, bilateral pleural effusion, soft tissue density shadow, and enlargement of lymph nodes in the mediastinum (Fig. 1a-d). The cardiac ultrasound revealed a large pericardial effusion with fibrous bands that could not be drained by ultrasound-guided puncture. Hemorrhagic fluid was aspirated from the bilateral pleural cavity. The chylous test of pleural fluid was positive. Positron emission tomography (PET) -CT showed diffuse interstitial thickening of the lungs, and high-density material resembling stipes and flocci at the root of the neck, bilateral clavicular regions and mediastinum (Fig. 1e). Slightly increased FDG uptake (SUVmax = 3.0) was found in the enlarged lymph nodes of the mediastinum, and diffuse reduction of the intensity in the C7, T1-T8, and T10 vertebral bodies and sacral vertebrae was also reported. A thoracoscopy was performed to confirm the diagnosis. Pathologically, the pleural biopsy showed thickening of the pleura with dilated lymphatic vessels. There was lymphoid tissue hyperplasia and lymphoid tissue infiltration by foam cells. An ultrasound-guided puncture biopsy of the stripe and floccus lesion situated in the neck was performed. Pathological examination of the neck mass biopsy showed proliferation of dilated lymphatic structures in the adipose tissue. Immunohistochemical staining of the spindle cells for actin was positive. Staining for HMB45 and melan-A was negative. These findings were consistent with lymphatic dilatation.

**Fig. 2:**
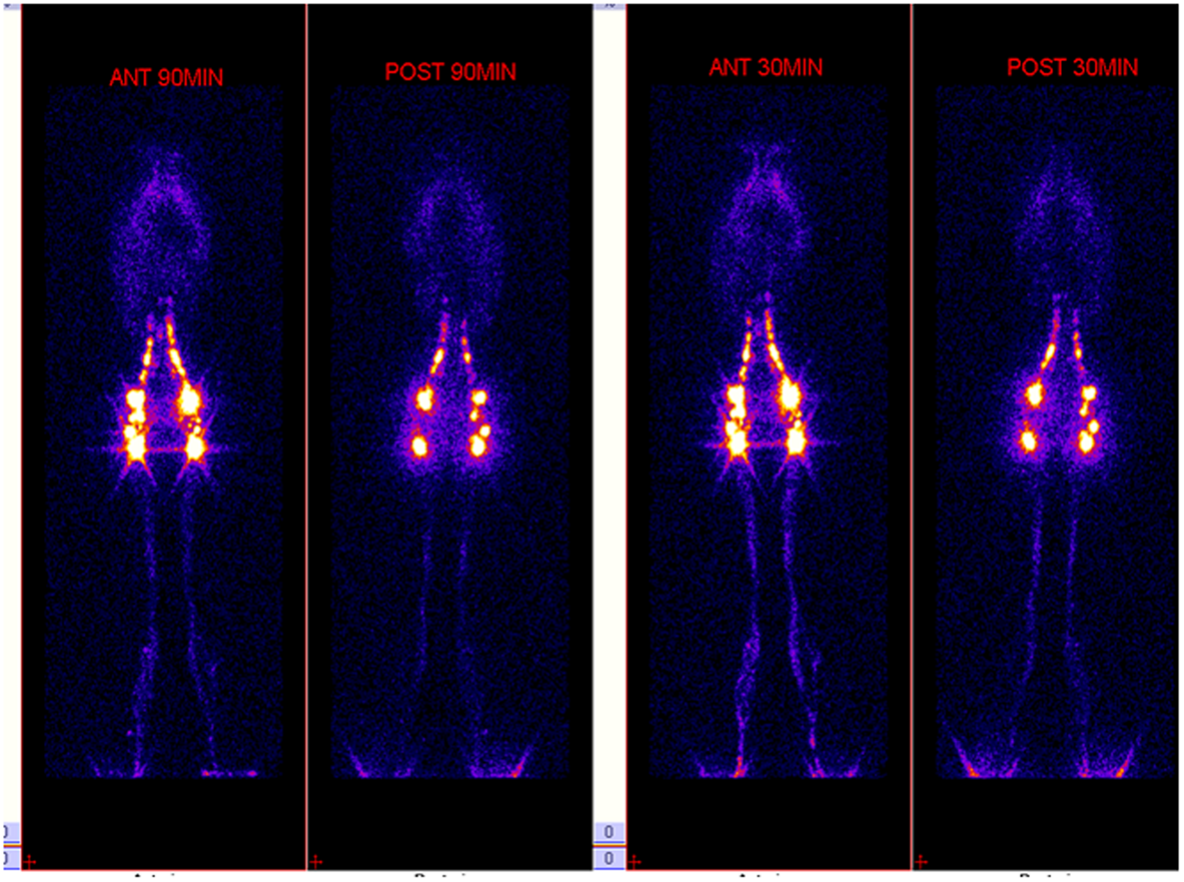
A Lymphangioscintigraphy (radionuclide lymphangiogram) of the chest images shows significant radioactive uptake in the chest, which was higher than the liver shadow. Lower limb lymphatic reflux is observed without signs of obstruction

The patient was formulated with a fat –free diet with the addition of medium-chain triglycerides. She also received a daily intravenous infusion of 10 g of albumin. After receiving the treatment described above, the patient continued to experience polypnea, palpitations, lower extremity edema, cachexia, and persistent hypoproteinemia. A lymphangioscintigraphy (radionuclide lymphangiogram) showed significant radioactive uptake in the chest, which was higher than the liver shadow (Fig. 2). The radioactive uptake by chest tissue was significantly increased, showing a mediastinal distribution, and was similar to that seen in the neck area with blood flowing through the large vessels. The lymphangiogram showed abnormal reflux of the bilateral chest lymphatic tube. Lymphatic dilatation or abnormal drainage could not be ruled out. Lower limb lymphatic reflux was observed without signs of obstruction.

**Fig. 3:**
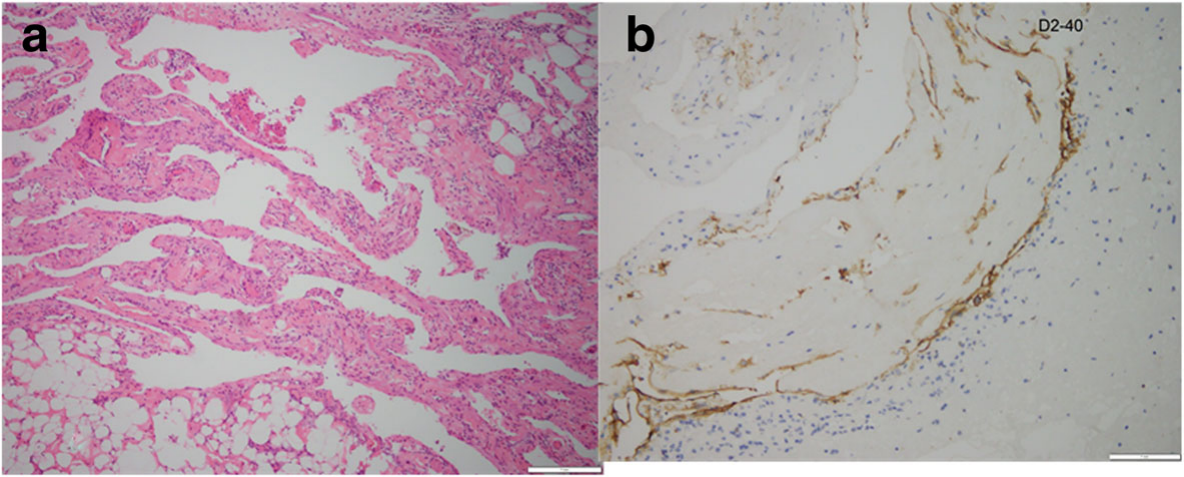
Pathological findings of the proliferating tissue during pericardiectomy. **a** Histologic analysis shows the proliferating tissue consisting of dilated lymphatic-like structures of various sizes. Spindle-shaped smooth muscle bundles are seen around parts of the lumen. (hematoxylin and eosin) (**b**) Immunohistochemical tests with the D2–40 antibody show that the abnormal lymphatic vessels stain positive for the lymphatic endothelial antigen recognized by this antibody

The patient had a pleural drainage for almost 2 months, with persistent fluid production of>300 ml/day. For further diagnosis and treatment, we did not use pleural sclerosing agents.She had aggravated breathing difficulties and uncontrollable hypoproteinemia. An echocardiogram revealed increased pericardial thickness, septal bounce, and limited diastolic function of heart. The peripheral venous pressure was 18.4 mmHg. The patient received a surgical pericardiectomy through a median sternotomy to treat CP. Intraoperatively, marked thickening of the pericardium and local edema was observed, and the thickness of the stripped pericardium was determined to be 5–8 mm. The pericardium was carefully stripped from the outflow tract all the way to the aortic root. Then, the space between the great arteries and the anterior wall of the superior vena cava was dissociated. Exploration revealed abundant hyperplasia of tubular tissue in the aortopulmonary window in both pleural cavities. The hyperplastic tissue was plexiform, like tortuous enlarged pipes with increased lymph fluid pressure. The proliferating tissue was completely resected and the pericardium was stripped. Histopathologic examination showed that the proliferating tissue consisted of dilated lymphatic-like structures of various sizes. Spindle-shaped smooth muscle bundles could be seen around parts of the lumen. Immunohistochemical tests with the D2–40 antibody showed that the abnormal lymphatic vessels stained positive for the lymphatic endothelial antigen recognized by this antibody (Fig. 3). After surgery, the patient’s symptoms subsided and her chylothorax gradually disappeared. One year later, she has no recurrent pleural effusion or pericardial effusion.

## Discussion and conclusion

The clinical course and intraoperative findings of this case are consistent with a diagnosis of GLA. The International Society for the Study of Vascular Anomalies (ISSVA) approved new guidelines for the classification of lymphatic disorders at the 20th ISSVA Workshop in Melbourne, Australia in 2014. Disorders of the pulmonary lymphatic system include macrocystic, microcystic, and mixed lymphatic malformations, GLA (formerly known as diffuse lymphangiomatosis), channel-type lymphatic malformations, lymphatic malformations in Gorham-Stout disease (GSD), and primary lymphedema [2]. GLA may affect superficial soft tissues, the skin, bone, and abdominal and thoracic viscera [3]. Lymphangiomas usually occur at the age of 2, but they may not be discovered until adulthood. Due to their scarcity, these latter situations are often misdiagnosed and their management is also difficult [4].

Although lymphangioma is a benign neoplasm, it rarely undergoes spontaneous remission and often progresses to cause serious morbidity and even death. Because of its slow growth,it is believed that the performance of thoracic lymphangiomas occurs after the incubation period. Thoracic lymphangiomas are still asymptomatic for many years and become apparent only when the patient has problems due to compression of vital structures. Thoracic lymphangiomas can cause pleural/pericardial effusions, compressive symptoms, or dyspnea [5].

In this report, GLA affected the neck, thorax, pericardium, and bone. Chylothorax and pericarditis were the chief symptoms. Our patient was an adult woman who had no symptoms in the first 28 years of her life. This particular case is interesting in many ways. Rapid progression of the disease, as observed in this patient, has never been reported previously. The patient showed uncontrollable hypoproteinemia. The cardiac surgery was performed to treat CP and persistent chylothorax. Histopathological analysis of the thoracoscopic biopsy specimens in this case could not distinguish the disease from other lymphatic disorders. Therefore, a PET-CT scan and multidisciplinary consultation were necessary for this patient with multiorgan involvement. Lymphangioscintigraphy helps evaluate lymphatic truncal anatomy [6]. The lymphangioscintigraphy and PET-CT results in this patient allowed us to exclude the presence of lymphatic obstruction and tumors. We strongly recommend lymphangioscintigraphy in cases of unexplained chlothorax, since it is a non-invasive means of identifying a lymphatic leak and the eventual presence of anatomic aberrations.

In our patient, proper diagnosis was difficult because of the overlapping findings and because it was easy to misdiagnose the previous pathological results. The differential diagnosis included lymphangioleiomyomatosis(LAM), lymphangiectasis and GLA. LAM was ruled out based on the negative staining with HMB45 and melan-A. Open- thorax surgery showed evidence of the characteristic proliferation and dilation of the lymphatic vessels. Intraoperatively, macroscopic findings and histologic examination of biopsy specimens confirmed the diagnosis of lymphangioma rather than lymphatic dilatation.

GLA is usually typically on the basis of clinical signs, imaging examinations, and histological findings. Open-lung biopsy may be considered the gold standard diagnostic test for GLA. The aim of medical management for chylothorax is to relieve respiratory symptoms by drainage of the pleural effusion, the treatment of underlying causes, and the prevention of, or treatment for malnutrition and immunodeficiency [7]. The treatment of problems associated with chylothorax involves chest-drainage, a medium –chain triglycerides diets, and administration of octreotide andsomatostatin. This patient did not show any improvement after conservative treatment. Finally, pericardial stripping surgery was performed, and hyperplastic lymphatic tissue found in the mediastinum and pleural cavity was resected. After surgery, the patient gradually improved.

In summary, we report a case of GLA with chylothorax and constrictive pericarditis in a 29-year-old woman. This patient exhibited remarkable features, including a continuously hemorrhagic chylothorax, constrictive pericarditis, and involvement of bone and neck lymph nodes. After attempting to manage her condition with conservative treatment, the patient underwent pericardial stripping surgery. Therefore, an open- lung biopsy and surgical intervention are necessary for the diagnosis and treatment of this condition.

## Abbreviations

CP: constrictive pericarditis
CT: computed tomography
GLA: Generalized lymphatic anomaly
LAM: lymphangioleiomyomatosis
PET: Positron emission tomography
